# Lupins and Health Outcomes: A Systematic Literature Review

**DOI:** 10.3390/nu14020327

**Published:** 2022-01-13

**Authors:** Lesley Bryant, Anna Rangan, Sara Grafenauer

**Affiliations:** 1 Nutrition and Dietetics Group, Sydney Nursing School, Faculty of Medicine and Health, Charles Perkins Centre, The University of Sydney, Camperdown, NSW 2006, Australia; lbry9477@uni.sydney.edu.au (L.B.); anna.rangan@sydney.edu.au (A.R.); 2 Grains & Legumes Nutrition Council, Mount Street, Sydney, NSW 2060, Australia; 3 School of Medicine and Health, University of New South Wales, Randwick, NSW 2052, Australia

**Keywords:** lupin, health outcomes, type 2 diabetes, cardiovascular disease, obesity

## Abstract

Lupins have a unique nutrient profile among legumes and may have beneficial health effects when included in the diet. The aim of this systematic review was to investigate the effects of lupin on a range of health outcome measures. Databases included MEDLINE, Embase and CINAHL, and focused on controlled intervention studies on healthy adults and those with chronic disease such as type 2 diabetes, cardiovascular disease and overweight. The Preferred Reporting Items for Systematic Reviews and Meta-Analyses protocol was followed. Investigated intervention diets utilised whole lupin, lupin protein or lupin fibre, and outcomes were measured by markers of chronic disease, body weight and satiety. Quality assessment of results was performed using the Cochrane revised risk of bias tool. Overall, 21 studies with 998 participants were included: 12 using whole lupin, four used lupin protein and five lupin fibre. Beneficial changes were observed in 71% of studies that measured blood pressure, 83% measuring satiety and 64% measuring serum lipids. Unintended weight loss occurred in 25% of studies. Whole lupin demonstrated more consistent beneficial effects for satiety, glycaemic control and blood pressure than lupin protein or lupin fibre. Heterogeneity, low study numbers and a small participant base indicated further studies are required to strengthen current evidence particularly regarding the protein and dietary fibre components of lupin.

## 1. Introduction

Lupin (*Lupinus*) is a legume of the Fabaceae family that has formed part of the human diet since early civilisations. Legumes such as chickpeas, lentils, peas, beans and pulses continue to be a staple food in many parts of the world. Prized by consumers for their highly nutritious and inexpensive nature, they are becoming increasingly valued by producers for their ecological sustainability. With an increasing awareness of the benefits of regular legume consumption to human health, particularly among people living with chronic disease, lupin may be a useful addition to the food supply. Australia accounts for approximately 85% of the world’s lupin production. It is grown predominantly in Western Australia, with some parts of New South Wales, Victoria and South Australia also under cultivation [1,2]. Lupin grows well in poor agricultural conditions, is pest-resistant and requires less water than many other food crops, therefore is ideally suited to Western Australia’s climate and sandy soils. Moreover, it helps to improve soil fertility by the nitrogen fixing action of its rhizome, a characteristic shared with all legume plants [3]. The two most common varieties grown are the narrow-leafed lupin, also known as Australian sweet lupin or blue lupin (*Lupinus angustifolius*) and the white or albus lupin (*Lupinus albus*). Species of lupin cultivated globally include *Lupinus mutabilis* and *Lupinus luteus* or yellow lupin.

Macronutrient profiles of the various lupin species differ slightly. Compared with other legumes, Australian sweet lupin has one of the highest combinations of both protein and fibre. While protein can make up as much as 40% and fibre 30% as dry weight, with an additional 5% inulin, its carbohydrate content accounts for less than 10% [4]. As with other legumes, lupins have a high nutrient density, yet their energy density is low. They are rich in minerals such as iron, magnesium, zinc, calcium and potassium; they contain vitamin A, B vitamins and vitamin E; and the fat profile predominantly consists of mono- and polyunsaturated fats, omega 3, 6 and 9 fatty acids [5]. Despite these benefits, Australians include very little lupin or other legumes in their diet, compared with populations in other countries. According to the Grains and Legumes Nutrition Council’s 2017 consumption study [5], only 28% of people in Australia eat legumes, a modest 4% increase in three years. Conversely, secondary analysis of the National Nutrition and Physical Activity Survey (NNPAS 2011-12) [6] suggests consumption was around 4 g per day, perhaps indicating use as a snack rather than as a staple food within meals. According to dietary modelling, Australians will need to eat almost five times more legumes to meet the Australian Dietary Guidelines recommendation [7]. Lupin is an excellent source of high-quality plant protein for people who follow a vegetarian or vegan diet. It is gluten-free and provides a more nutrient-dense wheat replacement than other grain and cereal alternatives currently utilised in gluten-free diets. The Australian food industry is beginning to recognise the value of lupin and a range of lupin products is now available, including whole lupin flakes, flour, crumb, meal, kibble and splits. The addition of lupin into other foods enhances their nutritional value and may be an acceptable approach to introduce lupin into the food supply, particularly as an ingredient [8]. Flour made from whole lupin can be easily incorporated into many foods and isolated protein and fibre from the lupin seed may also be of benefit, though separation of the component parts is a more complex process. Interest in lupin as a functional food is increasing among food manufacturers, however it is not clear whether there is a difference in biological effect between whole lupin, lupin protein and lupin fibre when consumed as part of the diet. Sensitivity to legume protein allergens may have an inhibitory effect on lupin consumption in some individuals. In 2017 Food Standards Australia New Zealand included lupin among allergens that must be declared on food products, however, allergic response to lupin is milder and occurs less frequently compared with exposure to peanut and soy [9]. According to Allergy and Anaphylaxis Australia, it is estimated that less than 1% of the Australian population is allergic to lupin [10].

Despite the increasing popularity of plant-based diets, it has been some years since a review of the health benefits of lupin intake was conducted. A 2015 review of the literature on lupins, among a broad range of other legumes, suggested *Lupinus angustifolius* may beneficially effect blood pressure, blood lipids, insulin sensitivity and the gut microbiome [8]. A 2016 review of the nutritional, chemical and health-promoting properties of *Lupinus albus* recognised its potential in the production of functional food [11]. A systematic literature review in 2020 on the effects of legume consumption on markers of glycaemic control in people with and without diabetes excluded studies of less than six weeks’ duration, therefore post-meal and short-duration lupin interventions were not captured [12]. The aim of this systematic review was to investigate the effects of lupin on a range of health outcome measures.

## 2. Materials and Methods

This systematic literature review was conducted in accordance with the Preferred Reporting Items for Systematic Reviews and Meta-analyses (PRISMA) guidelines [13]. The study protocol was submitted to the International Prospective Register of Systematic Reviews (PROSPERO) for registration (TBC).

### 2.1. Eligibility and Exclusion Criteria

To research the question ‘Is there an effect of lupin consumption on health outcomes in humans?’ a framework was developed using the PICO (Population, Intervention, Comparator/Control, Outcome) format (Table S1). Studies were eligible for inclusion in this review if they met the following criteria: (a) controlled intervention trial of any duration and of parallel or cross-over design; (b) populations comprising any adults aged 18 years and above, with or without chronic disease, overweight or obesity; (c) dietary interventions in the form of whole lupin, such as dried, pickled and brined seeds, flakes, flour, crumb, meal, kibble and splits, or components of lupin such as protein and fibre; (d) assessed the effect of lupin consumption on biomarkers of chronic disease such as any related to diabetes, cardiovascular disease, hypertension, hypercholesteremia, hyperlipidaemia, cancer, inflammation, and oxidative stress, or assessed the effect of lupin consumption on anthropometric measurements and perceptions of satiety in relation to overweight and obesity and their association with chronic disease risk. The exclusion criteria applied to the study search were: (a) participants below 18 years of age; (b) assessments of single isolated proteins, peptides or alkaloids from lupin; (c) lupin intake as a supplement in capsule form; (d) non-English language studies; (e) publication dates before 1 January 2000.

### 2.2. Search Strategy

The following databases were searched: EMBASE (via Ovid) MEDLINE (via Ovid), CINHAHL (via EBSCO) from 1 January 2000 until 13 September 2021. Reference lists of eligible studies were scanned and searched manually on PubMed for additional studies.

### 2.3. Study Selection, Data Extraction, and Quality Assessment

Search results were imported into EndNote X9^®^ referencing software (EndNote X9, Clarivate Analytics, Philadelphia, PA, USA) and duplicates were removed. Screening of studies was performed in two stages, first by title and abstract, then by full text. A data extraction form was created in Microsoft^®^ Excel^®^ spreadsheet (Microsoft 365 MSO Version 2109.14430.20306, Redmond, WA, USA) to include study citation, design, and duration; participant numbers and characteristics; intervention and control diet information; outcomes measured, and results obtained. The included studies were assessed for within-study risk of bias using the revised Cochrane risk-of-bias tool (RoB2) for randomised controlled trials [14]. Reviewer L.B. assessed studies to determine whether each study had low, some concerns, or high risk of bias. Areas of uncertainty were resolved in consultation with a second reviewer (S.G.). Assessment domains included risk of bias arising from the randomisation process, period and carryover effects, deviations from intended interventions, missing outcome data, measurement of the outcome, and selection of the reported result [14].

## 3. Results

### 3.1. Search Results and Study Selection

**Figure 1:**
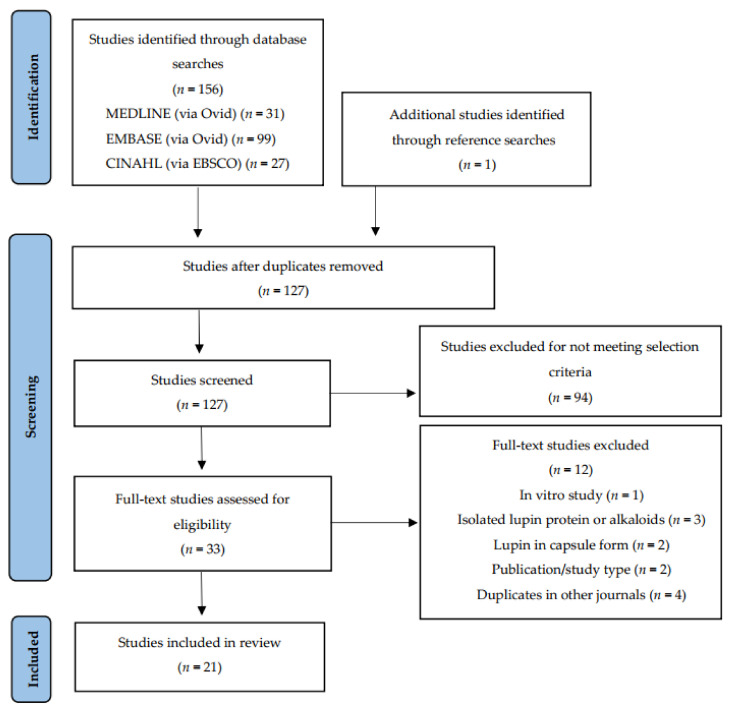
Preferred Reporting Items for Systematic Reviews and Meta-Analyses (PRISMA) flow diagram for study selection.

The search was conducted on 13 September 2021, returning a total of 157 records. One additional paper was identified from the reference lists of eligible studies. Following automated removal of duplicates by Endnote X9, 127 studies remained. Screening by title and abstract excluded 94 studies. Principal reasons for exclusion were publication type and study aim, interventions and measured outcomes beyond the scope of this review. A full-text review of the remaining 33 led to the exclusion of a further 12 studies. Reasons for exclusion were: duplications in other journals and/or non-English languages (*n* = 4), brief conference or workshop communications (*n* = 2), lupin intervention administration in capsule or supplement form (*n* = 2), investigation of lupin fractions such as alkaloids or single proteins (*n* = 3) and in vitro study protocol (*n* = 1). A total of 21 journal articles of controlled intervention studies met the inclusion criteria and were included in this qualitative review (Figure 1). Two studies generated three articles that reported on different sets of variables within each. These articles were treated as stand-alone studies and included in the final total.

### 3.2. Study Characteristics

Six of the included studies were randomised controlled trials (RCTs) of parallel design, the remaining 15 were cross-over studies, of which 11 were RCTs, while two were controlled, non-randomised, crossover studies. Research was based predominantly in Australia with smaller number of studies conducted in Germany, Italy and Ecuador. Table 1 lists study locations and the species of lupin under investigation. Five studies recruited healthy men and women [15,16,17,18,19], three recruited healthy men only [20,21,22], four studies involved men and women with type 2 diabetes [23,24,25,26], five with hypercholesterolaemia [27,28,29,30,31] and four involved people who were overweight or obese [32,33,34,35]. The number of completing participants ranged from *n* = 5 to *n* = 175 per study. Approximately 25% of the 998 participants across all studies were classified as healthy. Duration of study periods ranged from post-meal studies to 12 months. Categorisation of studies by form of lupin administered during treatment phases identified three distinct groups: whole lupin, lupin protein and lupin fibre. Study characteristics and outcomes of 12 whole lupin studies are summarised in Table 2, four lupin protein studies in Table 3, and five lupin fibre studies in Table 4. Measured outcomes were multiple and varied across studies, with *p* < 0.05 being declared as statistically significant.

### 3.3. Risk of Bias

**Figure 2:**
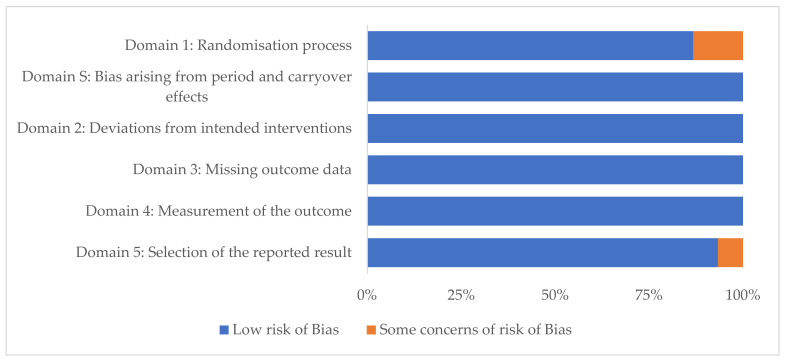
Within-study risk of bias assessment using the revised Cochrane risk-of-bias tool (RoB2) in 15 randomised (*n* = 11) and non-randomised (*n* = 2) controlled cross-over trials examining health outcomes of lupin consumption.

Each study was assessed according to the criteria outlined in the revised Cochrane RoB2 tool for RCTs. All parallel studies had a low risk of bias. Most cross-over studies had a low risk of bias (Figure 2); exceptions were one study with some concern for risk of bias in Domain1: Randomisation process, and in Domain 5: Selection of the reported result [25]. One other cross-over study had some concern in Domain 1 only [24].

### 3.4. Range of Investigated Health Measurements and Their Outcomes

**Figure 3:**
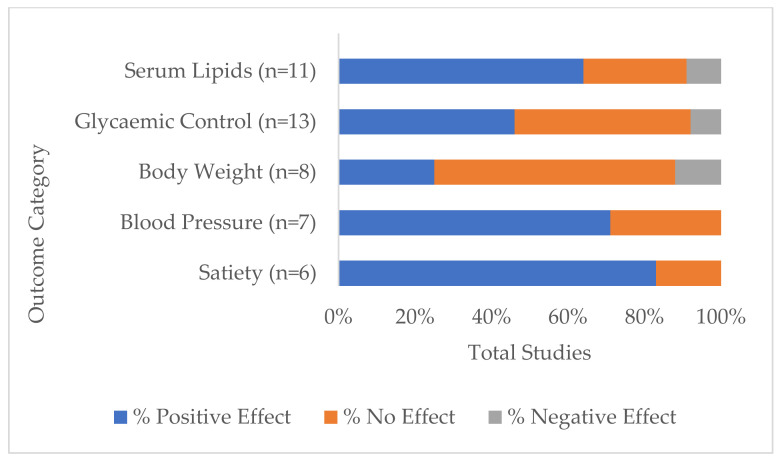
Percentage of total studies that reported differences between baseline and/or comparators (*p* < 0.05) by the five most investigated groups of health markers: serum lipids, glycaemic control, body weight, blood pressure and satiety, that had positive (desirable), negative (detrimental) or no effect on health outcomes.

The five most investigated variables or groups of variables across all studies and the direction of lupin consumption effect are shown in Figure 3. These were:

Serum lipids, which included any one or more of total cholesterol, LDL and HDL cholesterol, LDL:HDL ratio and triglycerides. Eleven studies measured serum lipids [19,22,23,25,27,28,29,30,31,34,35], of which 64% had one or more positive outcomes (i.e., showed statistically significant within-study difference(s) from baseline and/or control in a direction considered optimal for good health, depending on the health marker tested). Three studies did not report differences in serum lipids [19,23,34], while one study reported reduced HDL cholesterol with other lipids unchanged [35].

- Glycaemic control included measurements of post-prandial and long-term glucose and insulin levels, HOMA-IR and HbA1c. These were measured in 13 studies [15,16,17,18,22,23,24,25,26,27,28,34,35] with positive effects indicated in 46% of these studies [15,16,17,24,26,35].
- Body weight measurements were monitored in 8 studies [22,23,25,28,29,30,34,35], of which 25% recorded unintended weight loss [25,29], while 12.5% (one study) recorded increased body weight [30].
- Blood pressure was measured in 7 studies [23,25,29,30,31,33,35], two of which did not find significant differences [23,31] while the remaining five (71%) recorded reductions.
- Perceptions of satiety were monitored in 6 studies [16,17,18,22,24,29]. A significantly higher satiety rating score was given for lupin in 83% of studies, while one study saw no difference to control [22].

Variables measured in five or fewer studies included those relating to inflammation, such as hs-CRP [28,29,30,31,35], IL-6 and sICAM-1 [28], oxidative stress [32], and those relating to food intake [16,17,18,29], appetite, digestion and metabolism, such as concentrations of serum ghrelin [16], leptin [34], adiponectin [28,34], bowel function [19,21,24,29], faecal SCFA concentrations [19,21,29], faecal bile acid concentrations [19,29] and faecal microbiome varieties and populations [20]. No studies found significant differences in inflammation and oxidative stress markers, nor in the gastric hormones and adiponectin. However, bowel function changes were seen in all but one of the studies [24]. All studies that measured faecal SCFA concentrations and the faecal microbiome composition study found significant changes.

### 3.5. Range of Health Outcomes by Lupin Treatment Category

#### 3.5.1. Whole Lupin

**Figure 4:**
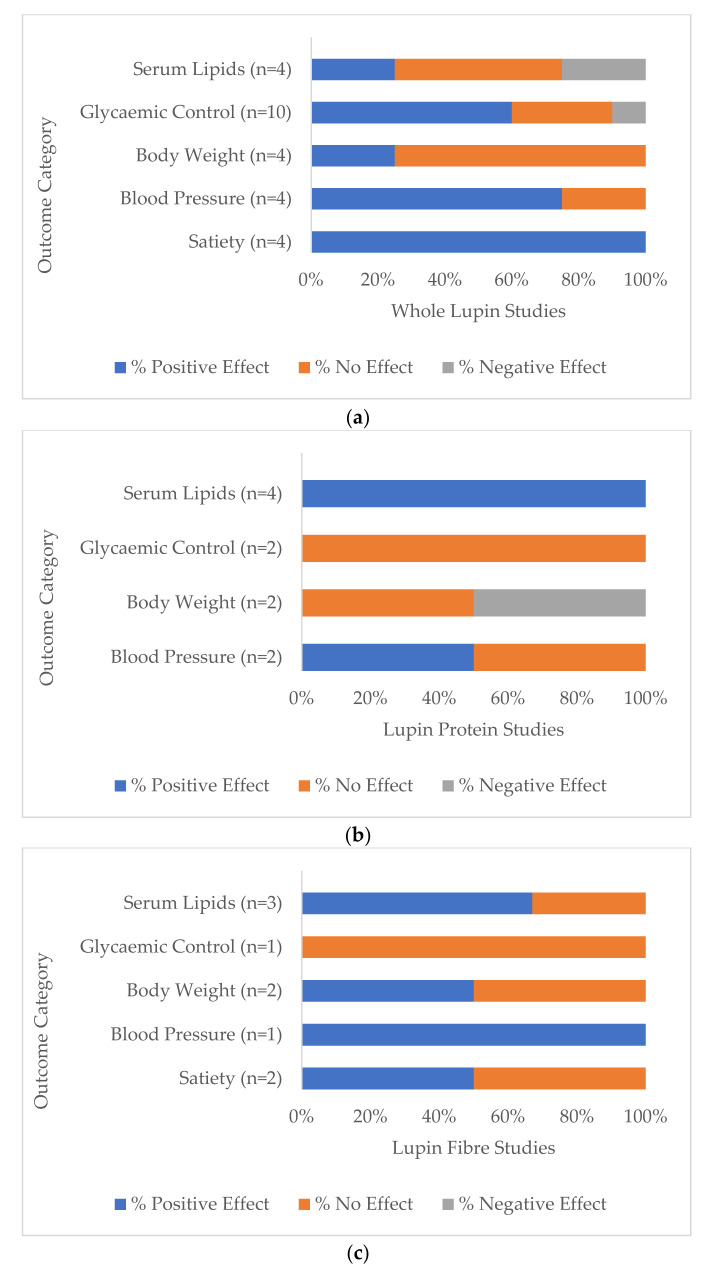
Percentage of total studies categorised by (**a**) whole lupin, (**b**) lupin protein and (**c**) lupin fibre treatment that reported positive (potentially beneficial), negative (potentially detrimental) and no significant differences between baseline and/or comparators (*p* < 0.05) in the five most investigated groups of health markers: serum lipids, glycaemic control, body weight, blood pressure and satiety.

Seventy-five percent (*n* = 9) of all whole lupin studies (*n* = 12) reported a significant difference in at least one of the health markers under investigation, compared to baseline or to the control group. While most of the directions of change were desirable or positive outcomes for that variable, one study of overweight and obese participants reported decreased levels of HDL cholesterol, thereby reducing its potential heart-protective benefit, while other lipid markers did not change [35]. One study [25] reported increased serum glucose at 14 and 28 weeks and increased insulin levels at 28 weeks following a daily lupin snack in the first phase and two lupin snacks per day in the subsequent phase. Key health outcomes for whole lupin are shown in Figure 4a. All whole lupin studies (*n* = 4) that measured perceptions of satiety reported desirable changes [16,17,18,24], as well as 75% (*n* = 3) of studies that monitored blood pressure (*n* = 4) [25,33,35] and 67% (*n* = 6) of studies that measured glycaemic control (*n* = 10) [15,16,17,24,26,35]. Serum lipids moved to healthier levels in 25% (*n* = 1) of studies (*n* = 4) [25], as well as reporting decreased body weight measurements [25].

#### 3.5.2. Lupin Protein

All lupin protein studies (*n* = 4) reported significant differences in at least one of the targeted health markers. All studies that measured serum lipids [27,28,30,31] and one study [30] of a total of two that measured blood pressure [30,31] reported significantly reduced levels (Figure 4b).

#### 3.5.3. Lupin Fibre

All lupin fibre studies (*n* = 5) reported significant differences in at least one of the measured health markers. Two out of the three lupin fibre studies that measured serum lipids reported significant differences and positive health outcomes [22,29], as well as the one study that measured blood pressure [29] (Figure 4c). All three lupin fibre studies that monitored bowel function reported positive changes [19,21,29].

## 4. Discussion

This systematic review of the evidence for health outcomes from lupin consumption observed a range of results across many biological and anthropometric health markers that variously resulted in no effects, positive effects and negative effects in terms of optimum health. In the 21 studies that met the selection criteria, the strongest evidence related to a lowering effect on total cholesterol, LDL cholesterol and LDL cholesterol:HDL cholesterol ratio; reduction in systolic blood pressure, increased satiety and improvement in post-prandial and glycaemic control. This supports the notion that lupin is equally and possibly more effective among all legumes in protecting long-term health. After categorising studies by form of lupin utilised in each intervention, this review noted potential relationships between lupins in their whole form and increased satiety perception, decreased blood pressure and improved glycaemic control, whereas lupin protein and lupin fibre demonstrated strongest positive results for blood pressure and serum lipids (though from a small study base).

These results correspond with the Kouris-Blazos et al. [8] review which concluded that sweet lupins may favourably effect blood pressure, blood lipids, insulin sensitivity and the gut microbiome. The Prusinski review [11] of white lupin concurred, stating that people who experience health conditions such as diabetes, hypertension, obesity, cardiovascular disease, hyperlipidaemia and colorectal cancer may benefit from the incorporation of this legume in the diet. However, research for the review centred on physiological properties of white lupin rather than on evidence for actual health outcomes. A 2017 review and meta-analyses investigating relationships between mortality and the intake of various food groups [36] found an inverse association between all-cause mortality and increased consumption of legumes, with no further dose response after 150 g/per day.

### 4.1. Whole Lupin

Nutrients in foods are metabolised in the human body according to the food matrix [37]. Categorisation of studies in this review by type of lupin administration, i.e., whole lupin, its protein and its fibre component, revealed greater health benefits were observed for the consumption of the whole food. Improved health outcomes were consistent for blood pressure [25,33,35], satiety [16,17,18,24] and glycaemic control markers [15,16,17,24,26,35] in whole lupin treatment studies, indicating benefits for reducing risk and managing symptoms of hypertension, cardiovascular disease, diabetes and obesity. Although it was noted that evidence for an increased satiety effect was present, evidence was less convincing for weight loss. None of the three whole lupin studies that measured body measurements detected a significant reduction in body weight [23,34,35]. Several reasons may account for this observation. Participants followed *ad libitum* diets that were not intended for weight loss, other lifestyle factors impacting weight, such as physical activity, were not monitored, and treatment duration may have been too short to demonstrate significant change. Hodgson et al. [34] proposed that if the observed trend in weight loss after four months was extended, a significant reduction of 2 kg could be expected within two years. The study further proposed that while *ad libitum* diets that are high in protein and dietary fibre may result in loss of body weight, the amount of protein and fibre in whole lupin may be a factor. In addition, the mostly insoluble fibre present in lupin may not be as effective as isolated soluble fibre used in many dietary fibre weight loss studies. Nevertheless, the broad health benefits proposed by whole lupin consumption suggest a synergistic interplay of macro- and micronutrient components within the whole food matrix and their influence on multiple biological functions [37], leading to improved long-term health outcomes.

### 4.2. Lupin Protein and Fibre Components

Protein and dietary fibre components of lupin individually demonstrated consistent evidence for lipid lowering effects [22,27,28,29,30,31]. These benefits were observed in hypercholesterolaemic participants, as well as in one of two studies that recruited healthy participants, yet whose average baseline total cholesterol was above 5 mmol/L [22]. Given that lipid levels did not change significantly in the lupin component study based on healthy men and women with average baseline total cholesterol below 5 mmol/L [19], this suggests lupin protein and fibre had moderating effects above this level. Evidence for blood pressure reduction from lupin component interventions with that of whole lupin does not conclusively favour its protein or fibre alone due to the paucity of studies. Serum lipids were measured by a similar number of studies across all three categories of lupin treatment. While results were variable, mostly positive effects were observed in diets that contained isolated lupin protein and lupin fibre. Substantially higher quantities of protein and fibre were provided in component trial protocols, compared with the amounts obtainable from the whole food in whole lupin studies. This may have contributed to the more consistently positive lipid outcomes in these lupin component studies. While the evidence for any health benefits from isolated lupin component consumption cannot be confirmed from so few studies, the addition of lupin protein to foods and beverages for maximising protein intake may be a useful alternative to soy and whey protein, particularly for consumers avoiding phytoestrogens and animal proteins. Similarly, lupin fibre is a gluten-free alternative for individuals with coeliac disease.

### 4.3. Dose Response

Indication of a dose response relationship between lupin and health outcomes was not identified due to multiple forms of lupin delivery and study methods among studies. However, one study [25] designed a dose response protocol comprising a doubled intake of whole *Lupinus mutabilis* during one intervention phase from 10 g to 20 g per day. While there was no change in glycaemic response markers between the two doses, blood pressure reduction was greatest after the increased dose phase.

### 4.4. Healthy vs. Unhealthy Participants Health Outcomes

Substantial evidence for health marker differences between healthy participants and those with type 2 diabetes, hypercholesterolaemia or who were overweight or obese, was unable to be determined because of the heterogeneity among studies. Having undertaken a sub-group analysis, however, one lupin fibre study [22] on generally healthy subjects found no significant effect on serum lipids among normocholesterolaemic participants (baseline total cholesterol < 5.5 mmol/L), while LDL cholesterol was significantly lowered among participants identified by study authors as ‘mild to clinically hypercholesterolaemic’ (baseline total cholesterol > 5.5 mmol/L). The investigation of whole *Lupinus mutabilis* consumption in type 2 diabetic subjects under conventional non-insulin medication found significantly reduced glycosylated haemoglobin (HbA1c) outcomes among a sub-group with less severe disease (HbA1c maintained at ≤ 8%), while no HbA1c effect was found in the remaining group that maintained HbA1C ≥ 8% and <10% [25]. Though changes in biomarkers may not be demonstrated consistently in healthy subjects, it can be supposed that lupin consumption offers protective benefits in hypercholesterolaemia and in well-controlled hyperglycaemia, if not in disease of greater severity.

### 4.5. Progression of Lupin and Health Outcomes Knowledge

Reflection on 16 years of lupin and health research revealed a progression from a focus on principal biomarkers for chronic disease, to a broader scope that encompassed other related health markers and possible biological mechanisms of effect. Analysis of faecal SCFA composition after lupin fibre consumption proposed that increased concentration and output of acetate and butyrate may have a protective effect on colorectal cancer risk [21]. A study on lupin fibre that observed a reduction in serum lipids in hypercholesterolaemic individuals proposed that increased bile acid excretion was not the result of bile acids binding to fibre, but a lower environmental pH from the fermentation of lupin fibre in the gut and the subsequent release of SCFAs [29]. Since the single study that focussed on lupin fibre and faecal gut bacteria was in 2006 [20], current interest and greater understanding of the gut microbiome warrants further investigation.

### 4.6. Strengths and Limitations

This systematic literature review was undertaken with the acknowledgement of several strengths and limitations. A major strength was the inclusion of high quality RCTs and non-randomised controlled studies that disclosed valid contextual reasons for non-randomisation. Implementation of the revised Cochrane risk-of-bias tool facilitated recognition and acknowledgement of any limitations within studies. Limitations within this review relate to potential publication bias as only research published in English language journals was targeted. Relatively few studies met the selection criteria and the participant base was limited. Furthermore, the objectives, methods and analyses of the various studies lacked homogeneity, thus precluding a meta-analysis to be performed.

### 4.7. Future Directions

The subject of lupin consumption and health outcomes is a relatively new area of investigation, therefore more research is required to expand the evidence base. This should comprise multiple studies with similar aims, designs and protocols based on adequately sized population groups. Studies should identify the species of lupin and test all lupin forms in quantities that could feasibly be included in a normal diet, preferably in a dose response manner. This would allow for a more accurate assessment of the evidence overall for health benefits and optimum intake. In terms of health outcome measures, those for blood lipids, blood pressure and glycaemic control would be the most useful in identifying the unique nutritional and physiological properties of lupin. Furthermore, studies that involve concurrent investigations on healthy populations and those with different degrees of disease severity will inform whether lupin consumption may be more useful as a risk-reduction strategy or in chronic disease minimisation.

## 5. Conclusions

This is the first systematic review to our knowledge to investigate the range of health outcomes and lupin consumption according to its mode of delivery, either as a whole food or the protein or fibre component. This review found divergent results in the effects of lupin consumption on many health marker outcomes, though greatest indications of benefit were apparent in improved satiety and reductions in blood pressure, and to a lesser extent in reductions in serum lipids and improved glycaemic control. More often, evidence was based on the whole lupin providing a broader range of health benefits than was observed in the smaller number of component studies. While the evidence for lupin’s health benefits is promising, more substantial research would be required before health claims could be made. Nevertheless, its unique nutritional and physiological properties, particularly as a whole food, make it an ideal legume to include in a healthy diet.

## Figures and Tables

**Table 1:** Study location, lupin species and form of lupin consumed in eligible studies investigating the health benefits of lupin consumption.

Country	Reference	Lupin Species/Common Name (NS = Not Stated)	Whole Seed (W), Protein Isolate (PI) or Fibre Isolate (FI)
Australia	Hall et al., 2005 [18]	*L. angustifolius*	W
	Hall et al., 2005 [22]	*L. angustifolius*	FI
	Smith et al., 2006 [20]	*L. angustifolius*	FI
	Johnson et al., 2006 [21]	*L. angustifolius*	FI
	Lee et al., 2006 [16]	NS	W
	Lee et al., 2009 [33]	NS	W
	Yang et al., 2010 [32]	NS	W
	Hodgson et al., 2010 [34]	*L. angustifolius*	W
	Dove et al., 2011 [26]	*L. angustifolius*	W
	Keogh et al., 2011 [17]	NS	W
	Belski et al., 2011 [35]	*L. angustifolius*	W
	Skalkos et al., 2020 [24]	Australian Sweet Lupin	W
	Ward et al., 2020 [23]	*L. angustifolius*	W
Germany	Weiße et al., 2010 [27]	*L. angustifolius*	PI
	Bähr et al., 2013 [30]	*L. angustifolius*	PI
	Fechner et al., 2013 [19]	*L. angustifolius* and *L. albus*	FI
	Fechner et al., 2014 [29]	*L. angustifolius*	FI
	Bähr et al., 2015 [31]	*L. angustifolius*	PI
	Schopen et al., 2017 [15]	*L. albus*	W
Italy	Sirtori et al., 2012 [28]	*L. angustifolius*	PI
Ecuador	Fornasini et al., 2019 [25]	*L. mutabilis*	W

**Table 2:** Characteristics and major outcomes of studies examining whole lupin consumption and health outcomes.

Reference	Study Type	Subjects (*n*) and Characteristics	Intervention	Control/Comparator	Energy Balance	Main Health Markers	Main Outcomes
Hall et al., 2005 [18]	RCT single blind cross-over Post-meal study	*n* = 11 Healthy men (*n* = 9) and women (*n* = 2). Mean age 31.6 years, range 25–45 years	Breakfast including lupin bread with 10% wheat flour replaced with Australian sweet lupin kernel flour	Breakfast including standard recipe white bread	95kJ difference in lupin breakfast (1338 kJ) and control breakfast (1243 kJ)	120 min SG, SI 180 min satiety response. Glycaemic index (GI), insulinaemic index (II) and satiety index (WB = 100). Energy intake from *ad libitum* buffet and during remainder of day.	↓ GI (*p* = 0.022) ↑ II (*p* = 0.046) Trend to lower SG at 30 min. Peak satiety at 10 min WB and 25 min lupin. Below baseline at 160 min for WB. Lupin did not reach zero within 180 min. No difference in SI, satiety response, satiety index, energy intake at *ad libitum* buffet, or energy intake during remainder of day.
Lee et al., 2006 [16]	RCT cross-over Study 1: 4 treatments 1 week apart	Study 1: *n* = 16 healthy men (*n* = 8) and women (*n* = 8). Mean age 58.6 ± 7.2 years. Mean BMI 31.3 ± 4.5 kg/m^2^	Lupin bread 40% total flour (24% final weight of bread) in 4 treatments: WB-WB/WB-lupin/lupin-WB/lupin-lupin	White bread breakfast and lunch	Isocaloric at breakfast, *ad libitum* lunch	Post breakfast 180 min satiety response. Total energy intake after *ad libitum* lunch.	↑ Satiety at breakfast for satisfaction and prospective consumption (*p* < 0.001, *p* < 0.001). ↑ Satiety at 180 min for fullness (*p* < 0.001), satisfaction (*p* < 0.001) and prospective consumption (*p* < 0.001). ↓ Energy intake at lunch after lupin breakfast (−488; 95% CI: −798, −178 kJ). ↓ Intrameal energy intake when lupin consumed at lunch(−1028; 95% CI: −1338, −727 kJ).
	Study 2: 2 treatments 1 week apart	Study 2: *n* = 17 healthy men (*n* = 11) and women (*n* = 6). Mean age 61.0 ± 5.6 years. Mean BMI 27.2 ± 4.3 kg/m^2^	Lupin bread 40% wheat flour replaced with lupin kernel flour	White bread	Isocaloric	Post breakfast 180 min plasma ghrelin, SG and SI	Altered ghrelin response (*p* = 0.04) ↓ 180 min plasma ghrelin (*p* = 0.009). Altered glucose response (*p* = 0.01) ↓ glucose AUC (*p* = 0.006) ↓ insulin AUC (*p* = 0.002).
Lee et al., 2009 * [33]	RCT parallel study 16 weeks 2 cohorts	*n* = 74 Overweight and obese men (*n* = 26) and women (*n* = 48) Mean age 59.0 ± 7.4/56.8 ± 8.5 years. Mean BMI 30.6 ± 3.6/30.5 ± 3.4 kg/m^2^.	Lupin bread 40% wheat flour replaced with lupin kernel flour (24% final weight bread) to replace usual carbohydrate-rich foods to ~15–20% usual energy intake.	White bread to replace normal bread intake and other carbo-hydrate-rich foods to ~15–20% usual energy intake	Isocaloric	24-h SBP, DBP, pulse pressure and heart rate	↓ 24-h SBP (*p* = 0.03) ↓ pulse pressure (*p* < 0.001). No difference in DBP, heart rate
Yang et al., 2010 * [32]	Paper refers to the Lee et al., 2009 study above	As above	As above	As above	As above	Plasma and urinary F2-isoprostanes, plasma 20-HETE, plasma and urinary nitrite and nitrate concentrates	No difference between groups
Hodgson et al., 2010 * [34]	Paper refers to the Lee et al., 2009 study above	As above	As above	As above	As above	BW every 2 weeks Body composition at 16 weeks TC, HDL, TG, LDL, SG, SI, HOMA-IR, plasma leptin and adiponectin, hs-CRP	No difference between groups
Dove et al., 2011 [26]	RCT cross-over study of 3 test sessions, 7–14 days apart	*n* = 24 type 2 diabetic men (*n* = 19) and women (*n* = 5). Mean age: 57 ± 6.6 years, range 44–66 years. Mean BMI: 30.9 ± 4.8 kg/m^2^	Beverage of 50 g glucose and 50 g lupin kernel flour	Beverage of 50% glucose (control), beverage of 50% glucose + soya protein and fibre isolates (comparator)	All beverages matched for total volume, carbohydrates and fat content lupin and soya matched for energy, protein and fibre	240 min SG, SI and C-peptide response	↓ 240 min SG response (*p* < 0.001), ↑ 240 min SI and C-peptide responses (*p* < 0.001) ↓ SI response compared with soya (*p* = 0.013). No difference in SG and C-peptide responses between lupin and soya
Keogh et al., 2011 [17]	RCT cross-over study	*n* = 20 Healthy men (*n* = 10) and women (*n* = 10) Mean age 29.4 years Range: 20.1–44.8 years BMI 21.8 kg/m^2^ Range: 18.4–24.8 kg/m^2^	Lupin Bread breakfast	White bread breakfast (control), wholemeal and seeds bread breakfast (comparator)	Isocaloric breakfast, *ad libitum* standardised food and beverage tray 120 min post breakfast	120 min satiety, PG and insulin response. Food and beverage weight, energy and macronutrient content 120 min post meal	↑ Fullness response for lupin (*p* < 0.01) and WSB (*p* < 0.05) ↓ glucose AUC for lupin and WSB (*p* < 0.001) ↓ insulin AUC for lupin and WSB (*p* < 0.001) ↓ weight of food and beverage for lupin and WSB (*p* < 0.05) ↓ energy and total fat consumed after WSB (*p* < 0.05). No difference in energy and macronutrient intake post lupin meal.
Belski et al., 2011 [35]	RCT double blind parallel study 12 months 2 cohorts	*n* = 131 *n* = 93 at 12 months Overweight and obese men (*n* = 68) and women (*n* = 63) Mean age 46.5 ± 10.1/46.7 ± 9.4. Mean BMI 31.3 ± 2.7/31.4 ± 2.8 kg/m^2^	Lupin kernel flour in bread, biscuits and pasta	Standard food products without lupin (matched for colour, taste, texture)	Isocaloric	4 and 12 month BW, body composition SBP, DBP, TC, HDL, LDL, TG, SG and SI, HOMA-IR and hs-CRP.	↓ 24-h SBP and DBP at 12 months (*p* < 0.05) ↓ HDL (*p* < 0.05) ↓ SI and HOMA-IR at 4 and 12 months (*p* < 0.05) No difference in TC, LDL, TG, SG, hs-CRP BW or body composition at 4 or 12 months. No difference in maintenance of body weight loss during weight maintenance period (4–12 months)
Schopen et al., 2017 [15]	RCT single blind cross-over study 3 test visits 24 h apart	*n* = 12 healthy men (*n* = 5) and women (*n* = 7). Mean age men 28 ± 3.67 years, women 26.86 ± 3.44 years. Mean BMI men 24.72 ± 2.3 kg/m^2^, women 20.92 ± 1.63 kg/m^2^	Sweet lupin flour in lunch meal of pasta and meat sauce (0.94 g lupin flour per kg of participant body weight)	Pasta and meat sauce lunch (reference meal), pasta and meat sauce lunch with whey protein (0.42 g per kg of participant BW)	Standardised breakfast, standardised test lunch. Lupin and whey meals matched for protein. Reference meal ~22% less kJ and ~50% less protein per kg of participant BW. All test meals similar in carbohydrate	180 min SG 180 min SI Post test meal	↓ SG AUC 0–60 min (*p* < 0.001) ↓ SG AUC 0–180 min (*p* = 0.030). No difference in SI
Fornasini et al., 2019 [25]	Controlled non-randomised single blind cross-over study (28 weeks) (One-group pretest-posttest design with double pretest)	*n* = 5 type 2 diabetic men (*n* = 19) and women (*n* = 32) under conventional non-insulin medication. Mean age 64.1 ± 11.1 years. Mean BMI 30.3 ± 4.5 kg/m^2^	Whole *Lupinus mutabilis* 10 g dehydrated lupin snack. One dose per day 7 weeks (following initial 14-week medication only period). 2 doses per day next 7 weeks	Usual diet and medication	N/A	At 14 and 28 weeks BW, SBP, DBP, SG, SI, HbA1c, TC, LDL, HDL, Uric acid, CRP	↑ SG and SI 0–28 weeks (*p* ≤ 0.05) ↑ HDL 0–28 weeks (*p* ≤ 0.05) ↓ BW (*p* = 0.015) and BMI (*p* = 0.009) 0–28 weeks ↓ SBP and DBP 0–28 and 14–28 weeks (*p* ≤0.05). No difference in HbA1c, TC, LDL, uric acid, CRP
Skalkos et al., 2020 [24]	Controlled non-randomised cross-over study 3 consecutive days, 1 treatment per day	*n* = 20 Post-surgical hospital patient men (*n* = 12) and women (*n* = 8) with type 2 diabetes. Mean age 74.3 ± 11.7 years. Mean BMI 30.7 ± 4.5 kg/m^2^	4 × lupin biscuit containing 20% lupin flour (2 at morning tea and 2 at afternoon tea) on day 1	4 × wholemeal spelt biscuit day 2 4 × Arnott’s Marie biscuit (standard hospital option) day 3	Lupin and spelt biscuits isocaloric (1590 kJ/100 g) and lower than Marie biscuit (1850 kJ/100 g). Higher protein, fat and fibre in lupin and spelt, and lower carbohydrate and sugar than Marie biscuit	CGM interstitial glucose pre- and 5 timepoints post-meal, bowel function (Bristol Stool Chart), hunger and fullness rating	↓ glucose after dinner following lupin biscuit (*p* < 0.001) No difference in 0–90 min glucose at breakfast, morning tea, lunch, and afternoon tea for all 3 treatments. More patients felt fuller between afternoon tea-dinner following lupin biscuit (*p* = 0.018). No difference in bowel function
Ward et al., 2020 [23]	RCT double blind cross-over study 1-week run-in period, 2 × 8-week treatment with 8-week washout period	*n* = 22 *n* = 17 completed, men (*n* = 14) and women (*n* = 8) with moderate-to-well controlled type 2 diabetes (HbA1c < 9%) Mean age 58 ± 6.6 years. Mean BMI 29.9 ± 3.5 kg/m^2^	Lupin-enriched foods replacing 20% of daily energy intake. Consumed every breakfast, lunch and at least 3 dinners per week. Average daily intake ~45 g lupin per day (12 g/d protein 10 g/d fibre)	Wheat-based control foods	Isocaloric	SG (at waking, 1 h post breakfast, immediately pre-lunch and 1 h post-lunch), SI, HOMA-IR, BW, BP, TC, LDL, TG, HDL, C-peptide	No difference between treatments. Borderline significant decrease in TG with lupin

* Part of one study; Abbreviations: Area under the curve (AUC); Body mass index (BMI); Blood pressure (BP); Body weight (BW); Continuous glucose monitor (CGM); Diastolic blood pressure (DBP); Glycated haemoglobin (HbA1c); High density lipoprotein cholesterol (HDL); Homeostasis model assessment of insulin resistance (HOMA-IR); High-sensitivity C-reactive protein (hs-CRP); Low density lipoprotein cholesterol (LDL); Plasma glucose (PG); Systolic blood pressure (SBP); Serum glucose (SG); Serum insulin (SI); Triglycerides (TG); Total cholesterol (TC).

**Table 3:** Characteristics and major outcomes of studies examining lupin protein consumption and health outcomes.

Reference	Study Type	Subjects (*n*) and Characteristics	Intervention	Control/Comparator	Energy Balance	Main Health Markers	Main Outcomes
Weiße et al., 2010 [27]	RCT double blind parallel study 10-day run-in, 6-week treatment	*n* = 56 *n* = 43 completed, moderately hypercholesterol-aemic (5.7–7.9 mmol/L) men (*n* = 25) and women (*n* = 31). Mean age 43.9 ± 11.8 years. Mean BMI 25.9 ± 4.5 kg/m^2^	Blue lupin protein isolate, 35 g in 2 snack bars per day	Casein protein (CP), 35 g protein in snack bars per day	Isocaloric	LDL:HDL, TC, LDL, HDL, TG, PG, mRNA SREBP-2, LDL receptor and HMG-CoA reductase	↓ LDL:HDL for lupin compared to CP (*p* < 0.05) ↓ 0–6 week TC and LDL in lupin group ↓ 0–6 week TC, HDL, and TG for CP group (all *p* < 0.05) No difference between groups. ↓ alanine and glycine after CP ↓ methionine after Lupin, and ↓ than CP (*p* < 0.05) ↑ SREBP-2 in Lupin group, but not CP ↑ LDL receptor and ↓ HMG-CoA reductase in both groups (*p* < 0.05) No difference between groups for all 3 mRNA outcomes
Sirtori et al., 2012 [28]	RCT double blind, parallel study 4-week run-in, 4-week treatment	*n* = 193 *n* = 175 completed, moderately hypercholesterol-aemic (TC > 2200 mg/L) men (*n* = 82) and women (*n* = 93). Mean age range 52.7 ± 12.4–55.3 ± 14.6 years. Mean BMI range 24.0 ± 2.0–25.6 ± 3.2 kg/m^2^	Blue lupin protein isolate/cellulose fibre combination added to 2 snack bars per day	Control: casein/cellulose. Comparators: lupin/cellulose; pea protein/cellulose; casein/oat fibre; casein/apple pectin; pea protein/oat fibre; pea protein/apple pectin	Isocaloric	TC, LDL, HDL, TG, SG, SI, HOMA-IR, BW, adiponectin, sICAM-1, IL-6, hs-CRP	↓ TC lupin/cellulose (*p* < 0.05) (Greatest reduction in TC (*p* = 0.0098) and LDL (*p* = 0.004) in pea/apple pectin treatment). No difference lupin/cellulose for LDL, HDL, TG, SG, SI or HOMA-IR (casein/cellulose, casein/apple pectin and pea/oat fibre all decreased SI and HOMA-IR (*p* < 0.05). Pea/oat fibre also decreased SG (*p* < 0.05)). No difference in adiponectin or inflammatory markers
Bähr et al., 2013 [30]	RCT double-blind cross-over study 8-week treatment, 4-week washout	*n* = 33 hypercholesterol-aemic (TC ≥ 5.2 mmol/L) men (*n* = 33) and women (*n* = 18). Mean age range 49.4 ± 13.9–49.7 ± 12.8 years. Mean BMI range 27.3 ± 5.4–28.8 ± 6.5 kg/m^2^	Blue lupin protein isolate (LPI) protein drinks, 25 g LPI per day	Milk protein isolate (MPI) protein drinks, 25 g MPI per day	Isocaloric	TC, LDL, HDL, LDL:HDL, TG, 4 and 8week BW, SBP, DBP, resting pulse, urea, hs-CR	↑ HDL at week 4 for LPI compared to MPI (*p* = 0.036) No difference between treatments for lipids ↓ LDL for both treatments at 4 weeks but not at 8 weeks (*p* ≤ 0.008) ↓ LDL:HDL for LPI (*p* = 0.022) Both treatments slight ↑ BW and body fat from 0–8 weeks (*p* ≤ 0.045) No difference between treatments ↓ SBP for both (*p* ≤ 0.014) ↓ DBP and resting pulse for LPI (*p* ≤ 0.044) No difference between treatments. No difference between treatments in hs-CRP and urea 0–4 or 0–8 weeks, ↑ urea 0–4 weeks for both treatments (*p* ≤ 0.001) with smaller increases 0–8 weeks *(p* ≤ 0.022)
Bähr et al., 2015 [31]	RCT double blind, cross-over 3-phase study 28 days treatment 6-week washout	*n* = 72 *n* = 68 completed, hypercholesterol-aemic (TC ≥ 5.2 mmol/L) men (*n* = 28) and women (*n* = 40). Mean age range 50.4 ± 19.2–59.8. ± 9.3 years. Mean BMI range 24.9 ± 5.0–27.6 ± 4.4 kg/m^2^	Blue lupin protein isolate, 25 g consumed daily in 4 food products.	Milk protein (MP) 25 g in 4 food products; MP foods plus 2.5 g/d arginine in capsule form (MPA). Placebo capsules added to LP and MP diets for blindness	Isocaloric	TC, LDL, HDL, LDL:HDL, oxidised LDL, TG, SBP, DBP hs-CRP, urea, uric acid, homocysteine	↓ LDL after Lupin compared with MP (*p* = 0.044) ↓ 0–28 d TC (*p* < 0.001), LDL (*p* < 0.01) and HDL (*p* < 0.001) after lupin and MPA ↓ TG (*p* < 0.05) after Lupin Increases in urea were smaller for Lupin (*p* = 0.004) and MP (*p* = 0.001) compared with MPA ↓ Uric acid (*p* < 0.01) after lupin ↓ homocysteine after lupin compared with MP (*p* = 0.001) and MPA (*p* = 0.004)

Abbreviations: Body mass index (BMI); Body weight (BW); Diastolic blood pressure (DBP)); High density lipoprotein cholesterol (HDL); Homeostasis model assessment of insulin resistance (HOMA-IR); High-sensitivity C-reactive protein (hs-CRP); Interleukin-6 (IL-6); Low density lipoprotein cholesterol (LDL); Plasma glucose (PG); Systolic blood pressure (SBP); Serum glucose (SG); Serum insulin (SI); soluble intracellular cell adhesion molecule-1 (sICAM-1); Triglycerides (TG); Total cholesterol (TC).

**Table 4:** Characteristics and major outcomes of studies examining lupin fibre consumption and health outcomes.

Reference	Study Type	Subject (*n*) and Characteristics	Intervention	Control/Comparator	Energy Balance	Main Health Markers	Main Outcomes
Hall et al., 2005 * [22]	RCT single blind cross-over study 28 days of treatment 28 days washout period	*n* = 44 *n* = 38 completed, healthy men. Mean age 41.0 ± 1.9 years. Mean BMI 26.7 ± 0.5 kg/m^2^	Australian sweet lupin kernel fibre in foods within prescribed diet. 55 g dietary fibre/day for diets >9 MJ/day, 35 g dietary fibre/day for diets ≤9 MJ/day	Prescribed control diet without added lupin fibre. 25 g dietary fibre/day for diets >9 MJ/day, 18 g dietary fibre/day for diets ≤9 MJ/day	Isocaloric	TC, HDL, TG, PG and insulin, HOMA-IR, satiety perception, BW	↓ TC, LDL, TC:HDL and LDL:HDL for both treatments (*p* < 0.05) ↓ TC (*p* = 0.001), LDL (*p* = 0.001) TC:LDL (*p* = 0.006 ) and LDL:HDL (*p* = 0.003) for lupin relative to control. No difference in HDL and TG. No difference in PG for lupin (↓ PG in control (*p* = 0.001)) No difference in PG, insulin, HOMA-IR or satiety perception between treatments. No difference in BW for either treatment
Smith et al., 2006 * [20]	Paper refers to the Hall 2005 study above	*n* = 18 (randomly selected from above study)	As above	As above	As above	Measures of (i) total cells, (ii) total bacteria, (iii) *E. rectale*-*C. coccoides*, (iv) *Bacteriodes-Prevotella*, (v) *Enterobacteriaceae*, (vi) *C. histolyticum*/*C. lituseburense* group, (vii) *Lactobacillus-Enterococci*, (viii) *Bifidobacterium*, (ix) *C. ramosum*, *C. spiroforme* and *C. cocleatum* group	↑ *Bifidobacteria* (*p* = 0.001) ↓ *C. ramosum*, *C. spiroforme* and *C. cocleatum* group (*p* = 0.039) in lupin diet. No difference between treatments in total cells, total bacteria or populations of other species. Strong trend (*p* = 0.53) towards decreased *Bacteroides-Prevotella* in lupin diet
Johnson et al., 2006 * [21]	Paper refers to the Hall 2005 study above	*n* = 38 healthy men. Mean age 41.0 ± 1.9 years. Mean BMI 26.7 ± 0.5 kg/m^2^	As above	As above	As above	Frequency and ease of bowel motion, flatulence level, Bristol Stool Form, frequency (events), output, transit time, pH, faecal moisture content SCFA (total, acetate, propionate, isobutyrate, butyrate, isovalerate, valerate)	↑ Frequency (*p* = 0.047), ↑ faecal output (*p* = 0.020), ↓ transit time (*p* = 0.012), ↑ perception of flatulence level (*p* < 0.001), ↓ faecal pH (*p* < 0.001), ↑ faecal moisture content (*p* = 0.027), ↑ total SCFA concentration (*p* = 0.001) and ↑ daily output (*p* < 0.001), ↑ acetate concentration (*p* < 0.001) and ↑ daily output (*p* < 0.001) ↑ butyrate concentration (*p* = 0.006) and output (*p* = 0.002) ↑ valerate output (*p* = 0.030) with no difference in concentration. No difference in proprionate, isobutyrate or isovalerate.
Fechner et al., 2013 [19]	RCT double blind cross-over study 4 periods of 2 weeks each: run-in, 2 treatments and washout	*n* = 76 healthy men (*n* = 21) and women (*n* = 55). Mean age 24.4 ± 3.2 years. Mean BMI 21.7 ± 2.4 kg/m^2^	Blue lupin kernel fibre and white lupin kernel fibre. Total dietary fibre per treatment 25 g/d in beverages	Citrus fibre as active comparator for 2 lupin and 1 soya fibre treatments	Isocaloric	TC, HDL, LDL, TG, faecal pH, transit time, Bristol Stool Form, faecal SCFAs and bile acids	No change in serum lipids for all treatments, ↓ faecal pH for blue lupin (*p* < 0.01), no difference relative to citrus. ↓ Transit time, ↑ Bristol Stool Form score for blue lupin (*p* ≤ 0.05) ↑ Total SCFA, acetate, propionate and *n*-butyrate excretion for blue lupin (*p* ≤ 0.05). ↑ Primary bile acid excretion (*p* = 0.02) for blue lupin, ↓ total bile acid excretion for blue lupin relative to citrus. ↓ Total bile acid excretion for white lupin from run-in. ↓ Secondary bile acid excretion for blue and white lupin from run-in (*p* ≤ 0.05).
Fechner et al., 2014 [29]	RCT double blind cross-over study 3 intervention periods of 4 weeks each, run-in and 2 washout periods of 2 weeks each	*n* = 52 moderately hypercholesterol-aemic (TC >5.2 mmol/L) men (*n* = 20) and women (*n* = 32). Mean age: 46.9 ± 3.2 years. Mean BMI: 26.5 ± 5.9 kg/m^2^	Blue lupin kernel fibre 25 g/d	Citrus fibre 25 g/d as active comparator; control diet (CD) with no added fibre	Isocaloric	General excretion markers, faecal concentration or excretion of neutral sterols, bile acids and SCFAs. BW, body composition, BP, TC, HDL, LDL, TG LDL:HDL hs-CRP, satiety score	↓ Faecal pH from baseline (*p* ≤ 0.01) and against CD (*p* ≤ 0.001), ↓ transit time against CD (*p* ≤ 0.05), no difference in neutral sterols. ↑ Primary bile acids from baseline (*p* ≤ 0.05), no difference in total or secondary bile acids. ↑ Formation of total SCFA from baseline (*p* ≤ 0.001) and against CD (*p* ≤ 0.01), ↑ acetate from baseline and against CD (*p* ≤ 0.001), ↑ propionate from baseline (*p* ≤ 0.001) and against control (*p* ≤ 0.05), ↑ butyrate from baseline (*p* ≤ 0.01) and against control (*p* ≤ 0.05). ↓ BW, BMI, and WC from baseline (*p* ≤ 0.001) and against control (*p* ≤ 0.01). ↓ TC (9%), LDL (12%) and TG (10%) for lupin compared with citrus (*p* ≤ 0.02), ↓ hs-CRP (*p* = 0.02), SBP (*p* = 0.01) for lupin compared to baseline. ↑ Perception of satiety (*p* ≤ 0.001)

* Part of one study; Abbreviations: Body mass index (BMI); Blood pressure (BP)); Body weight (BW); High density lipoprotein cholesterol (HDL); Homeostasis model assessment of insulin resistance (HOMA-IR); High-sensitivity C-reactive protein (hs-CRP); Low density lipoprotein cholesterol (LDL); Plasma glucose (PG); Short-chain fatty acid (SCFA); Triglycerides (TG); Total cholesterol (TC).

## Supplementary Materials

The following are available online at https://www.mdpi.com/article/10.3390/nu14020327/s1, Table S1: PICO (Population, Intervention, Comparator/Control, Outcome) framework to define the search strategy for the question: ‘Is there an effect of human lupin consumption on health outcomes?’, File S2: Search terms (MEDLINE).
