# Supplementary material for: Lupins and Health Outcomes: A Systematic Literature Review
Source: Nutrients. 2022 Jan 13;14(2):327. doi: 10.3390/nu14020327 (PMC8777979; doi:10.3390/nu14020327)
Supplement: Supplementary file 1 [file nutrients-14-00327-s001.zip › nutrients-1536365-sup.pdf]

Supplementary Materials

Table S1. PICO (Population, Intervention, Comparator/Control, Outcome) framework to define the search strategy for the question: 'Is there an effect of human lupin consumption on health outcomes?'

PICO Categories	Inclusion Criteria	Exclusion Criteria
**Participants**	Any adults ≥18 years, with or without chronic disease	Non-human participants<br>Children aged <18 years
**Study type**	Controlled intervention trials, of any duration, parallel or cross-over design	Any other study type
**Interventions**	Consumption of whole lupin, including dried, pickled and brined seeds, flakes, flour, crumb, meal, kibble and splits. Lupin protein. Lupin fibre.	Provision of single proteins isolated from lupin, peptides, alkaloids or other fractions. Diet interventions that include lupin with other legumes. Non-dietary intake of lupin in capsule form.
**Comparators/Control**	Consumption of an alternative food or a regular diet	No comparator or control in study design
**Outcome**	Changes between baseline and follow-up and differences between treatments in markers of chronic disease, cancer, anthropometric measurements, and feelings of satiety	Qualitative measures other than those stated in the inclusion criteria

File S2. Search terms (MEDLINE)

1. 1.Adult/
2. adult*.mp
3. Humans/
4. participant*.mp.
5. Population/
6. population*.mp.
7. Persons/ or people.mp.
8. Male/ or m#n.mp.
9. Female/ or wom#n.mp.
10. Individual*.mp.
11. 1 or 2 or 3 or 4 or 5 or 6 or 7 or 8 or 9 or 10
12. Lupinus/
13. lupin*.mp.
14. sweet lupin*.mp.
15. white lupin*.mp.
16. albus lupin*.mp.
17. Lupinus albus.mp.
18. L albus.mp.
19. Lupinus angustifolius.mp.
20. L angustifolius.mp.
21. narrow-leafed lupin*.mp.
22. Lupinus mutabilis.mp.
23. Lupinus luteus.mp.
24. (lupin adj2 flake*).mp.
25. lupin kernel flour.mp.
26. lupin flour.mp.
27. (lupin adj2 kibble*).mp.
28. (lupin adj1 kibble*).mp.
29. (lupin adj1 crumb).mp.
30. (lupin adj2 crumb*).mp.
31. (lupin adj split*).mp.
32. lupin meal.mp.
33. lupin bean*.mp.
34. lupin seed*.mp.
35. lupin enriched diet*.mp.
36. (lupin* adj3 rich adj3 diet*).mp.
37. (lupin adj3 bread*).mp.
38. lupin kernel fibre.mp.
39. lupin fibre.mp.
40. lupin protein.mp.
41. lupin protein isolate*.mp.
42. Lupinus luteus.mp.

43. 12 or 13 or 14 or 15 or 16 or 17 or 18 or 19 or 20 or 21 or 22 or 23 or 24 or 25 or 26 or 27 or 28 or 29 or 30or 31 or 32 or 33 or 34 or 35 or 36 or 37 or 38 or 39 or 40 or 41 or 42
44. Randomized controlled trial*.mp.
45. Random allocation.mp.
46. double blind method.mp.
47. single blind method.mp.
48. RCT*.mp.
49. clinical trial*.mp.
50. cross over stud*.mp.
51. cross-over stud*.mp.
52. 44 or 45 or 46 or 47 or 48 or 49 or 50 or 51
53. Diabetes Mellitus/ or diabetes.mp.
54. (disease* adj2 marker*).mp.
55. diabet*.mp.
56. Type 2 diabet*.mp.
57. Type 1 diabet*.mp.
58. (T2D or T1D or NIDDM).mp.
59. glyc?em*.mp. or Glycemic Control/ or Blood Glucose/
60. metabolic syndrome.mp. or Metabolic Syndrome/
61. Insulin Resistance/ or insulin resistence.mp.
62. prediabet*.mp.
63. Blood Glucose/ or blood sugar level*.mp.
64. blood glucose level*.mp.
65. Glycated Hemoglobin A/ or HbA1c.mp.
66. insulin.mp.
67. insulin resistance.mp.
68. Cardiovascular Diseases/ or CVD.mp.
69. cardiovascular disease*.mp.
70. Hypertension/ or hypertension.mp.
71. blood pressure.mp. or Blood Pressure/
72. BP.mp.
73. Coronary Disease/ or Cholesterol/ or serum cholesterol level*.mp. or Hypercholesterolemia/
74. blood cholesterol level*.mp.
75. LDL cholesterol.mp.
76. HDL cholesterol.mp.
77. total cholesterol.mp.
78. Triglycerides/ or triglyceride*.mp. or Hypertriglyceridemia/
79. (blood or serum lipid*).mp.
80. cancer.mp.
81. Inflammation/ or inflammat*.mp.
82. C-reactive protein.mp. or C-Reactive Protein/
83. Body Mass Index.mp. or Body Mass Index/
84. body weight.mp. or Body Weight/

85. weight maintenance.mp.
86. Obesity/ or Obes*.mp.
87. Overweight.mp. or Overweight/
88. weight loss.mp. or Weight Loss/
89. (satiety adj2 level*).mp.
90. satiation.mp.
91. (full* adj2 feeling).mp.
92. early satiety.mp.
93. early satiation.mp.
94. satiety response.mp.
95. satisf*.mp.
96. Microbiota/ or microbiota.mp.
97. gut microbiome.mp. or Gastrointestinal Microbiome/
98. gut health.mp.
99. gut flora.mp.
100. gastrointestinal microbiome.mp.
101. 53 or 54 or 55 or 56 or 57 or 58 or 59 or 60 or 61 or 62 or 63 or 64 or 65 or 66 or 67 or 68 or 69 or 70 or 71 or 72 or 73 or 74 or 75 or 76 or 77 or 78 or 79 or 80 or 81 or 82 or 83 or 84 or 85 or 86 or 87 or 88 or 89 or 90 or 91 or 92 or 93 or 94 or 95 or 96 or 97 or 98 or 99 or 100
102. 11 and 43 and 52 and 101
103. limit 102 to (english language and yr="2000 -Current")
104. limit 103 to humans
